# A pH-Dependent Coarse-Grained Model for Disordered Proteins: Histidine Interactions Modulate Conformational Ensembles

**DOI:** 10.1021/acs.jpclett.4c02314

**Published:** 2024-09-09

**Authors:** Rivka Calinsky, Yaakov Levy

**Affiliations:** Department of Chemical and Structural Biology, Weizmann Institute of Science, Rehovot 76100, Israel

## Abstract

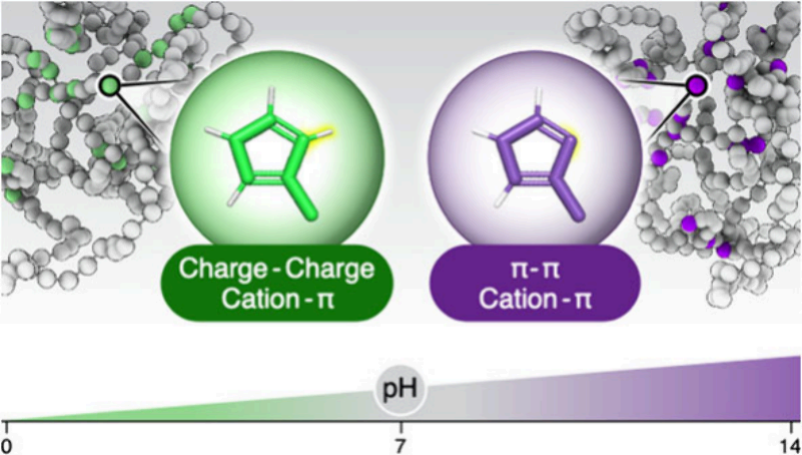

Histidine (His) presents a unique challenge for modeling disordered protein conformations, as it is versatile and occurs in both the neutral (His^0^) and positively charged (His^+^) states. These His charge states, which are enabled by its imidazole side chain, influence the electrostatic and short-range interactions of His residues, which potentially engage in cation−π, π–π, and charge–charge interactions. Existing coarse-grained (CG) models often simplify His representation by assigning it an average charge, thereby neglecting these potential short-range interactions. To address this gap, we developed a model for intrinsically disordered proteins (IDPs) that accounts for the properties of histidine (H). The resulting IDPH model is a 21-amino acid CG model incorporating both His charge states. We show that interactions involving previously neglected His^0^ are critical for accurate modeling at high pH, where they significantly influence the compaction of His-rich IDPs such as Histatin-5 and CPEB4. These interactions contribute to structural stabilizations primarily via His^0^–His^0^ and His^0^–Arg interactions, which are overlooked in models focusing solely on the charged His^+^ state.

Histidine (His) stands out as a relatively rare amino acid, comprising only about 2% of ordered and disordered protein sequences. This scarcity hints at the potential significance of incidences of protein enrichment with His residues, especially the occurrence of protein segments containing clusters of His (His-clusters). Uniquely, the p*K*_a_ value of His (p*K*_a__His_ = 6.3, for isolated free residue^1^) lies within the range of physiological pH values.^2^ The p*K*_a_ of His often exhibits high sensitivity to the surrounding protein sequence,^3,4^ which can lead to significant variations in its effective p*K*_a_ within different proteins. Consequently, His readily switches between its charged (protonated) state (His^+^, pH < p*K*_a_) and neutral state (His^0^, pH > p*K*_a_) in response to dynamic changes in local physiological pH. This pH-dependent behavior enables His to engage in diverse interactions, including electrostatic,^5^ cation−π,^6^*π–π*, and hydrogen-bonding interactions, as well as in metal binding.^7^ Some of these interactions are limited to specific His protonation states. For example, electrostatic interactions occur only with His^+^ and metal binding occurs solely with His^0^. However, His can form cation−π interactions either as a cation (*i*.*e*., His^+^ state) or as a π system (*i*.*e*., His^0^ state). The nature and strength of these interactions can significantly influence protein structure and can be modulated by a change in pH, thus regulating function. Recent studies even link variations in His protonation states to neurodegenerative diseases such as Alzheimer’s^8^ and to prion protein misfolding.^9^ Interestingly, His-rich proteins are overrepresented among those related to nervous system development.^10^ A prime example is an RNA-binding protein that engages in sequence-specific binding to the cytoplasmic polyadenylation element (CPE), namely, the His-rich neuronal protein CPEB4, where a mere eight-residue mistranslation is linked to idiopathic autism spectrum disorder (ASD).^3,11^

Intrinsically disordered proteins (IDPs),^12−15^ which lack a stable tertiary or secondary structure, are known for being enriched in charged and polar residues.^12^ These proteins sample a pool of conformations that exhibit deviations from those of typical random coil polymers. Since histidine’s interactions can vary with pH due to resulting fluctuations in its charge state, it might be a key player in these conformational changes.^16−20^ While recent research has explored the importance of π–π and cation−π interactions in disordered protein stability,^21,22^ the involvement of His, which is capable of acting as both a cation and a π system, is often overlooked.^23,24^

The fluctuating charge state and low natural abundance of His often lead to simplification of its representation in computational models, in which His is typically modeled in a single state, reflecting the average of its two protonation states. In commonly used coarse-grained (CG) models for predicting IDP conformations, His is assigned an averaged +0.5 e electrostatic charge^25^ (although variations such as +0.375 and 0 e have also been reported^23^). This approach biases the interactions of His residues toward negatively charged amino acids (aspartate, Asp; glutamate, Glu) and away from positively charged ones (arginine, Arg; lysine, Lys). It also underestimates the electrostatic strength of a fully protonated His residue.

Furthermore, these generalized representations focus primarily on electrostatic interactions directly affected by His protonation states. However, they neglect short-range interactions with other amino acids, which can be significant. For instance, His^0^ can interact with positively charged Lys and Arg residues through cation−π interactions (Figure 1), whereas His^+^ would be electrostatically repulsive. Additionally, His can interact with the same aromatic partners (phenylalanine, Phe; tyrosine, Tyr; tryptophan, Trp) in both its protonated and deprotonated states. The former interaction involves cation−π interactions, whereas the latter is considered a simple π–π contact. Consequently, uncertainty in defining the His protonation state extends to these different short-range interaction types and to histidine’s preferred partner residues. A recent theoretical study using quantum calculations^2^ revealed that His can participate in cation−π interactions as either the cationic or π species, pairing with different partners for each type of interaction (Phe, Tyr, or Trp for the former, Lys or Arg for the latter, as shown in Figure 1) depending on its protonation state. The strength of these interactions is comparable to and can even exceed those of other amino acids, suggesting their potential importance. For example, we found the strength of the His^0^–Phe (π–π) interaction to be comparable to that of an average Phe–Phe π–π interaction, whereas the strength of the His^+^–Phe interaction (cation−π) surpassed that of the commonly discussed cation−π interaction of Phe–Arg.^2^

**Figure 1:**
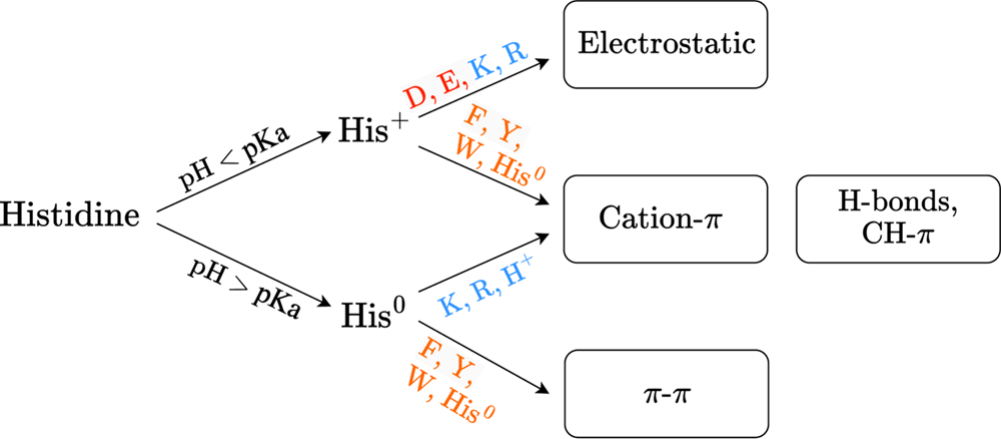
The pH-dependent interactions of the histidine residue. The protonation state of His under physiological conditions is sensitive to pH, and therefore, its most populated state is His^+^ at pH < p*K*_a_ or His^0^ at pH > p*K*_a_. Histidine’s protonation state determines the nature of the interactions it can engage in (electrostatic, cation−π, hydrogen bonding (H-bonds) or π–π), the role it plays in those interactions (cation or H-donor, when in the form His^+^; H-acceptor or π system when in the form of neutral His^0^), and the range of partner residues available to it in each context, as shown. Red font indicates negatively charged residues, blue font indicates positively charged residues, and orange font indicates aromatic residues.

The current study addresses the under-representation of His cation−π and π–π interactions in IDPs in existing CG models. We found that these contributions are essential to explaining the dependence of the dimensions of different His-rich peptides on the His content. To quantify the pH-dependent contribution that His interactions make to IDP conformations, we present a refined CG model of IDP dimensions that takes the properties of histidine (H) into account. The resulting IDPH model includes 21 types of amino acids and both His protonation states, His^0^ and His^+^, such that IDP proteins can be studied at both high and low pH values. The inclusion of both His protonation states enables the study of His interactions with various amino acids and includes not only different electrostatic contributions but also short-range cation−π and π–π interactions modeled through a Lennard-Jones-like potential. The refined parametrization of the IDPH model can both capture the dimensions of a small His-rich peptide and provide insights into the effect of pH on the conformational ensemble of the disordered region of the larger neuronal CPEB4 protein.

To quantify the effect of the pH-dependence of inter-residue interactions involving His on IDP dimensions, we studied inter-residue interactions under conditions of high and low pH, assuming that all His residues in a given sequence are either neutral or positively charged. Since most experimental observations are provided at pH ∼7.5, we focused primarily on the contributions of His^0^, namely, the His^0^–His^0^ (π–π) and His^0^–Arg (cation−π) interactions and the adequately represented His^0^–Phe/Tyr/Trp (π–π) interactions, as discussed in Methods.

*The IDPH Model Reproduces the Dimensions of Experimentally Studied His-Inclusive IDPs, Indicating Potential Role of His at High and Low pH*. Given that His has a p*K*_a_ of 6.3,^1^ it is expected that many His residues are in the neutral state in most healthy body tissues, and indeed, the Henderson–Hasselbalch relation estimates that 93% of His residues are in the His^0^ state under physiological conditions of pH ∼7.4. While the p*K*_a_ of specific His residues in proteins can vary depending on their local environment (e.g., solvent accessibility and neighboring residues), a recent survey^2^ of His p*K*_a_ has shown that the majority of His residues in structured proteins have a p*K*_a_ below 7.3. This supports the assumption that His can be reliably modeled as neutral under physiological conditions.

Consequently, it may be inappropriate to use CG models in which His residues possess an average electrostatic charge of 0.375 or 0.5 e to model IDPs at physiological pH values. To assess the IDPH model’s comparative performance in capturing IDP dimensions, we determined the radii of gyration (Rg) as calculated by the IDPH and Mpipi (His charge is fixed at 0.375 e) models and as found experimentally via small-angle X-ray scattering (SAXS; see the Supporting Information (SI), Table S1). We undertook the comparison at pH 7 (*i*.*e*., within the physiological range) on 16 His-inclusive sequences with a His content of 1–29% (see Figure 2A). Two additional IDPs (*i*.*e*., Tat and GRDBD94) were studied experimentally at lower pH and were modeled accordingly using the IDPH. Overall, the values from both models exhibited good correlations with the experimental values. Furthermore, better agreement was obtained between experimental and computed Rg values when using the IDPH model (mean squared error, MSE = 13.3 Å^2^) compared with the Mpipi model (MSE = 18.5 Å^2^), possibly because of model-dependent differences in the charge on His (1.0 and 0.375 in the IDPH and Mpipi models, respectively) and consequent increases in the QM-calculated strength of His^0^–His^0^ (π–π) and His^0^–Arg (cation−π) interactions (see Methods). To evaluate the energetic contributions of His to Rg, two control models (the Control IDPH and Control Mpipi) were designed (see the SI). In the Control IDPH model, His charge was set to a constant zero value at pH > pKa or +1 charge at pH < pKa, and the energetic terms for His^0^–His^0^ and His^0^–Arg, as defined in the original IDPH model, were turned off. This model produced Rg values with MSE = 15.0 Å^2^, thus illustrating the role of these short-range interactions with His at high pH. In the Control Mpipi model, the His charge used in the original Mpipi model was neutralized (*i*.*e*., reduced from 0.375 to 0.0). This model produced Rg values with MSE = 18.8 Å^2^, which is only slightly worse than the error value associated with the original Mpipi model, consistent with His electrostatics playing a minor role in the Mpipi model.

**Figure 2:**
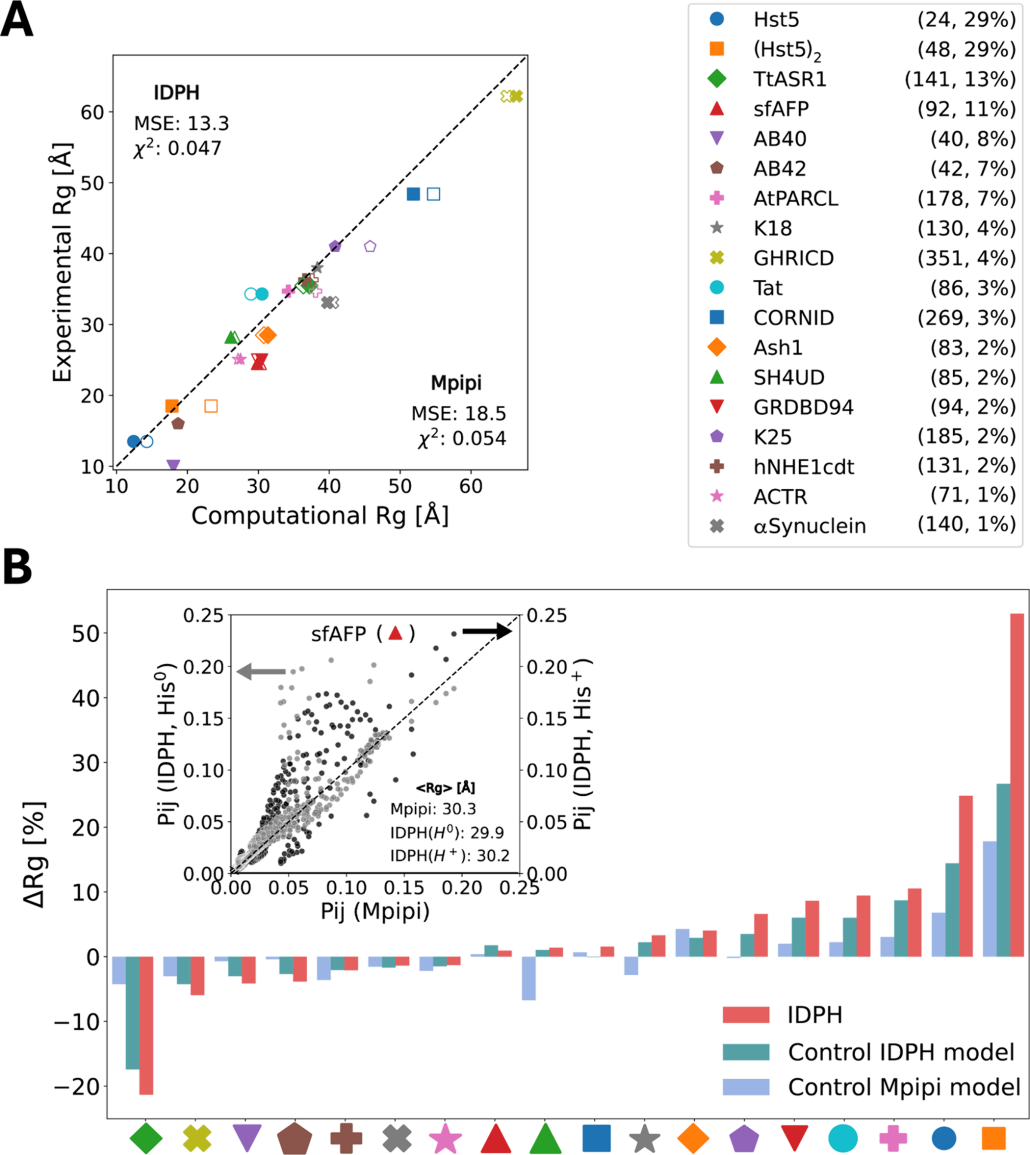
Performance of the IDPH and Mpipi models in capturing the dimensions of His-inclusive IDPs. (A) Correlation between the experimentally and computationally determined Rg values of 18 His-inclusive proteins (see SI Table S1 for values and errors). Each IDP is represented by a different symbol, and its length (in amino acids) and His content (%) are indicated. The Rg of each IDP was simulated using the IDPH and Mpipi models (filled and empty symbols, respectively). (B) The effect of pH change on the change in Rg, ΔRg = [[Rg_pH<7_ – Rg_pH>7_]/Rg_pH>7_], calculated for the 18 His-inclusive IDPs using the IDPH (red bars), Control IDPH (green), and Mpipi (blue) models. In all cases, His residues are neutral at high pH and charged at low pH (with a charge of +1 in the IDPH and control IDPH models and a charge of +0.375 in the Mpipi and Control Mpipi models). In the Control IDPH model, the unique terms of the IDPH model (*i*.*e*., His^0^–His^0^ and His^0^–Arg interactions) are turned off, and in the control Mpipi model all short-range interactions are adopted from the Mpipi model. The inset plots the correlation between the probabilities for specific pairwise contacts, Pij, in the sfAFP IDP as calculated by the IDPH and Mpipi models. The Pij values calculated by the IDPH model employing His^0^ (high pH; gray scatter) or His^+^ (low pH; black scatter) are mapped against the Pij values predicted by the Mpipi model for the same ij pair. Mean Rg values are noted for each model separately for comparison.

To investigate the effect of pH changes on the Rg of IDPs, we simulated the 18 His-inclusive IDPs using the IDPH model at both high and low pH values, which we defined as pH > 7 and pH < 7, respectively, to reflect a physiological pH range at which His deprotonation to His^0^ (when pH > p*K*_a_) and protonation to His^+^ (when pH < p*K*_a_) can occur. Figure 2B (red bars) shows the diverse changes in Rg (ΔRg(%) = [[Rg_pH<7_ – Rg_pH>7_]/Rg_pH>7_]) arising from changing His from His^0^ (in a high-pH environment) to His^+^ (in a low-pH environment), with 7 of the 18 simulated IDPs exhibiting compaction (ΔRg < 0) upon the transition from high to low pH and the other 11 simulated IDPs undergoing expansion (ΔRg > 0). For most of the studied IDPs (14 out of 18), the change in Rg with pH change was relatively small (|ΔRg|<10%); however, for some IDPs (mostly those with high His content), ΔRg was much higher, up to 50% (Figure 2B). To evaluate the molecular origin of the effect of pH change on ΔRg, we also calculated ΔRg using the Control IDPH and Mpipi models by neutralizing the charge on His upon switching from low to high pH. The ΔRg values calculated by the Control IDPH model (Figure 2B, green bars) were consistent with those obtained from the IDPH model, yet in some cases, they were much smaller. The comparison indicates that relying solely on electrostatic interactions (as in the Control IDPH model) is insufficient to capture the effect of pH on His and, particularly, that the full IDPH model’s inclusion of energetic terms for His^0^–His^0^ and His^0^–Arg interactions at high pH is an important addition to consideration of histidine’s electrostatic interactions. The ΔRg values obtained from the Control Mpipi model (Figure 2B, blue bars) were quite small (and in some cases even of opposite sign compared with those obtained using the IDPH model), indicating minimal changes to IDP dimensions with pH. This is not unexpected as the Mpipi was not originally designed to capture pH effects.

To further estimate the role played by His in modulating the conformational ensemble of IDPs, we examined the intramolecular interactions of an IDP whose calculated mean Rg values were similar when simulated using the IDPH and the Mpipi models. This scenario is evident in the His-rich sfAFP protein (∼11% His), which has ⟨Rg⟩ ≈ 30 Å according to both the IDPH (for either His^0^ and His^+^) and Mpipi models. However, despite their calculating similar dimensions, the underlying interactions differed significantly between the models, as can be inferred from the correlation plot (Figure 2B, inset) of the contact probabilities (Pij) between residue pairs for a comparison of IDPH values compared with the Mpipi values at high pH (gray dots) and at low pH (black dots). While most contacts showed minimal change, some interactions had different probabilities depending on the model used (see Supporting Information Figure S3). Thus, even for sequences where the two models agreed regarding an IDP’s averaged dimensions, the IDPH model provided additional insights into its biomolecular interactions.

*Previously Underestimated His^0^–His^0^ and His^0^–Arg Interactions Help Regulate the Dimensions of the His-Rich Disordered Peptide Histatin 5*. The large pH-induced change in the Rg of the (Hst5)_2_ peptide, which comprises two chained Hst5 peptides (ΔRg ≈ 50%, Figure 2B) indicated that, for this His-rich IDP, His^0^–His^0^ (π–π) and His^0^–Arg (cation−π) interactions contribute significantly to the conformational ensemble. To investigate whether the IDPH model reliably identifies the effect of these interactions, we focused first on the disordered Histatin 5 (Hst5, DSHAKRHHGYKRKFHEKHHSHRGY).^26−28^ This peptide was chosen because experimental Rg values^29^ are available for its wild-type (WT) and for five variants differing in their His content or in its distribution (Figure 3A, sequences 1–6, with WT Hst5 as sequence 5). Variants were produced by replacing some His residues with glutamate (Q), which is similar in size and also capable of participating in hydrogen bonds.

**Figure 3:**
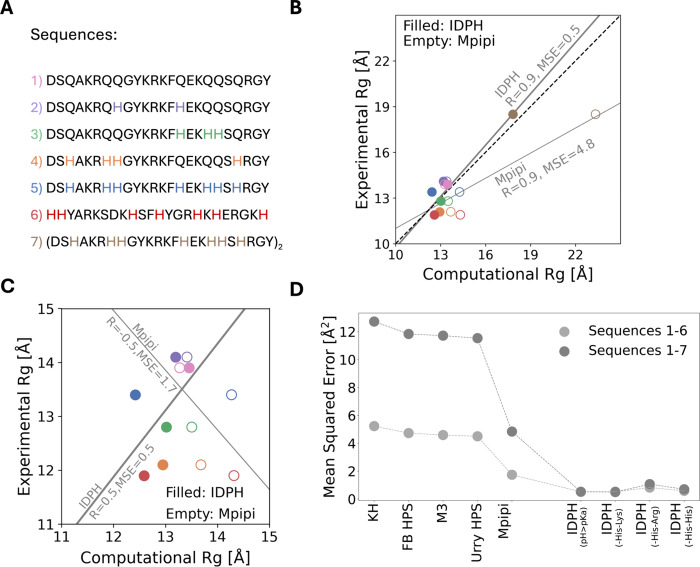
Performance of the IDPH model in capturing the dimensions of variants of the His-rich disordered protein Histatin5. (A) The seven studied variants of Histatin 5 ordered by the number of His residues. Variant 5 corresponds to wild-type Histatin 5, and variant 7 consists of two chained variant 5 peptides and is denoted (Hst5)_2_. (B) Correlation between the experimentally and computationally determined Rg values of Histatin 5 variants 1–7 (values and errors can be found in the Supporting Information Table S1). The computational Rg values were obtained from the IDPH (filled symbols) and Mpipi (empty symbols) models, with their correlations to the experimental Rg values indicated by thick and light gray lines, respectively, and the quality of the correlations quantified by the mean squared error (MSE) and *R* correlation coefficients. The black dashed line corresponds to perfect agreement *y* = *x* between the computational and experimental Rg values. (C) As per panel B but limited to Histatin 5 variants 1–6. (D) The MSE values calculated by comparing correlations between the Rg values of Histatin 5 variants 1–7 (dark gray circles) and 1–6 (light gray circles) as simulated by a range of models with the experimental Rg values. Coarse-grained models for intrinsically disordered proteins: KH; FB-HPS; M3; Urry HPS; Mpipi, multiscale π–π model; and IDPH, intrinsically disordered proteins including histidine properties model, applied as a full model under basic conditions ((IDPH (pH > 7)) and following the removal of His^0^–Lys (cation−π) interactions ((IDPH (−His–Lys),; His^0^–Arg (cation−π) interactions ((IDPH (−His–Arg)), or His^0^–His^0^ (π–π) interactions ((IDPH (−His–His)).

Notably, for variants 1–6, it was found that the value of Rg decreases as His content increases (see Supporting Information Figure S4). However, the distribution of His for a given His content is also important (refer to sequences 5 and 6). This suggests that His interactions contribute to the structures of Hst5 variants, making them more compact by increasing inter-residue interactions. Thus, WT Hst5 is a potential model peptide to study the overlooked contribution of His^0^–His^0^ and His^0^–Arg interactions to IDP dimensions. In addition, we considered the experimental Rg reported^30^ for (Hst5)_2_ (Figure 3A, sequence 7), which is of particular interest, as previous computational efforts failed to capture its Rg, presumably because interactions involving His resides were modeled as being weaker than interactions involving its counterparts: Phe, Tyr, and Trp.

The computational Rg values obtained by using the Mpipi CG model substantially deviate from the experimentally reported values (Figure 3B). Only sequences 1 and 2 (having no His or two His residues, respectively) showed agreement between the computational and experimental results. The Mpipi model consistently overestimated Rg values for most of the Hst5 sequences (Figure 3A), especially those with a higher His content (MSE = 4.8 Å^2^, Figure 3B). This overestimation was also observed when the Rg was calculated using other CG models such as the KH (MSE = 12.7 Å^2^) and FB-HPS (MSE = 11.8 Å^2^) models (Figure 3D). These observations suggest that current models either lack interactions that govern the size of these variants or at least inaccurately represent them.

Our implementation of His^0^–His^0^ (π–π) and His^0^–Arg (cation−π) interactions within the IDPH model (Figure 3B,C) significantly improved the prediction of Rg values for all variants (MSE ≈ 0.5 Å^2^), including both the single-sequence variants (sequences 1–6) and the longer chained (Hst5)_2_ variant (sequence 7), compared with values calculated using the Mpipi model. These findings highlight the potential importance of these interactions in governing the structures of His-rich peptides.

To verify that the improvement resulted from including these short-range interactions, rather than from charge–charge modifications, we tested Histatin variants using our Control IDPH model (see Supporting Information Figures S5 and S6). The Control IDPH model predicted only a weak correlation between the calculated and experimental Rg values for variants 1–6, which had similar calculated Rg values (Rg ≈ 13.5 Å) despite being characterized by different numbers or distributions of His residues (Supporting Information Figure S6). Overall, these results suggest that modifying solely the charge–charge contribution of His is insufficient to obtain accurately calculated Rg values for His-containing peptide sequences.

To identify the His^0^ interactions responsible for the loss of correlation between computational (Control IDPH) and experimental Rg values (see Supporting Information Figure S6), we separately removed either His^0^–His^0^ or His^0^–Arg interactions from the IDPH model. In both cases, elimination impaired the fit between the computational and experimental data (Figure 3D). Elimination of the His^0^ cation−π interaction led to a complete loss of correlation between experimental and computational Rg values, as the same calculated Rg value was then obtained for all variants (Supporting Information Figure S7A). Interestingly, removing His^0^–Lys (cation−π) interactions had minimal impact on the results (MSE ≈ 0.5 Å^2^) (Figure 3D and Supporting Information Figure S7B).

Although calculations of the Rg of Hst5 at high pH using the IDPH model assume that all His residues are neutrally charged, it is possible that some are actually transiently protonated and therefore should be modeled as His^+^ rather than as His^0^. We explored this scenario by examining the effect that mutating the first His of each Hst5 variant from His^0^ to His^+^ had on the Rg calculated by the IDPH model (Supporting Information Figure S8). While the MSE values remained comparable to those found when the Rg was calculated under the assumption that the entire His population was in the His^0^ state (Figure 3D), some variants benefited from this representation (particularly WT Hst5 and (Hst5)_2_ (sequences 5 and 7, respectively). However, for other variants, agreement with the experimental data weakened. This finding underlines the complex and variable influence of His on IDP dimensions.

*The IDPH Model Captures the pH-Dependent Contributions of Histidine’s π–π and Cation−π Interactions to IDP Dimensions*. To understand how pH affects stabilizing interactions in Hst5, we compared the contact maps generated by the IDPH and Mpipi models for sequence 6, which has the highest His content (29%), under conditions of both high and low pH. At high pH, the IDPH model predicts a higher frequency of contacts rich in both cation−π and π–π interactions compared with the Mpipi model (Figure 4A), with many of these contacts occurring between His and either aromatic or basic residues. The representation of His cation−π and π–π interactions in the IDPH model allows for the formation of previously overlooked conformations that are stabilized by these interactions (Figure 4B). The formation of stabilizing His interactions is coupled with an increased probability of the formation of additional favorable interactions. Figure 4A illustrates that cation−π and π–π interactions involving Phe (e.g., Phe–Phe and Phe–Arg interactions) are more populated when Hst5 is modeled with the IDPH model compared with the Mpipi model, despite the identical representation of contact strength in both models. We conclude that the refinement of His interactions in the IDPH model can increase the instances of stronger or comparable interactions involving other residues, even though these non-His-containing pairs were already adequately represented in the Mpipi model.

**Figure 4:**
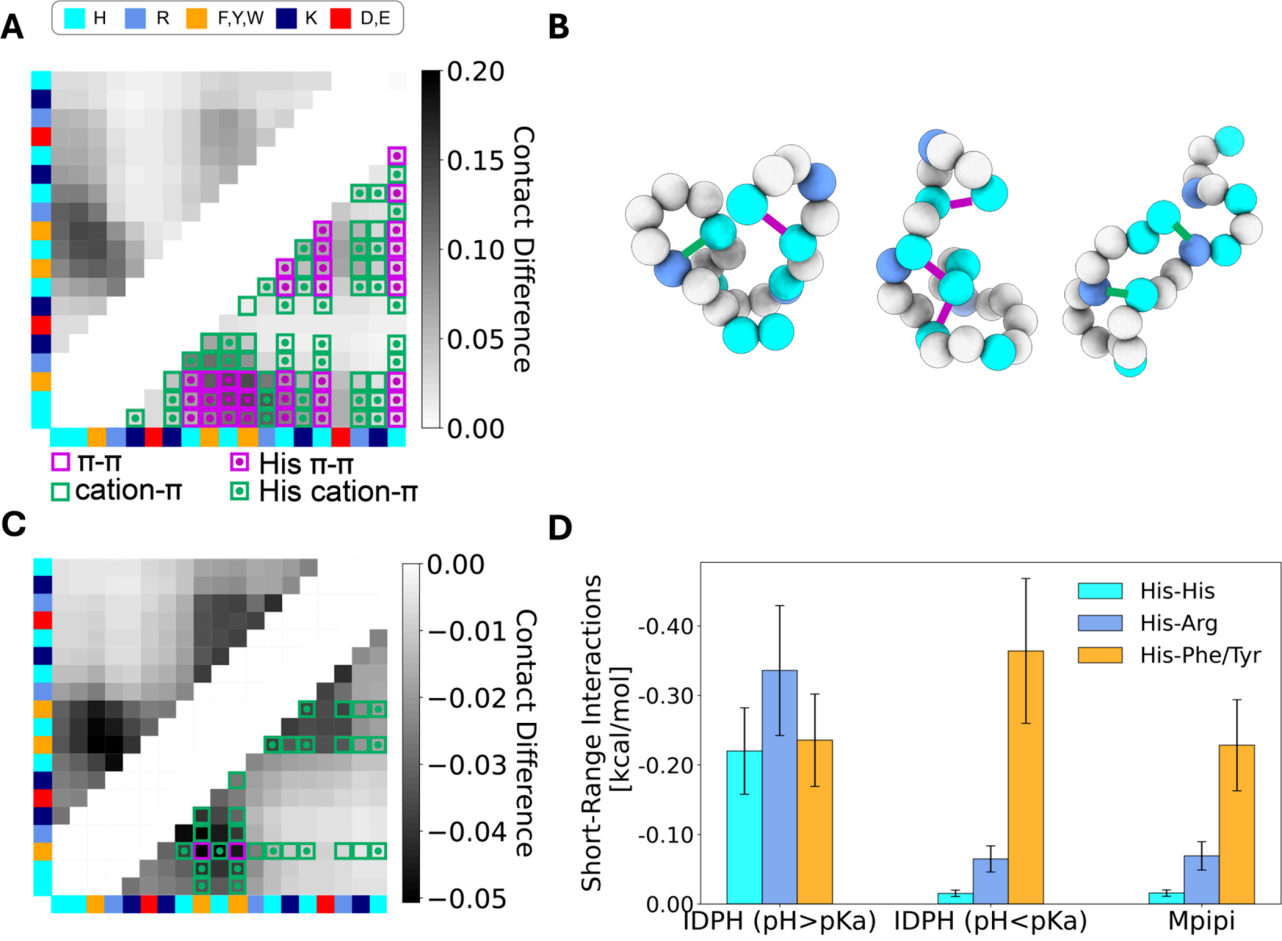
The population of cation−π and π–π interactions involving histidine in disordered proteins. (A) Changes to the map of inter-residue contacts within Histatin 5 (variant 6) as simulated by the IDPH (pH > 7) model referenced against the Mpipi model. The upper left trigonal shows the difference in contact probability (in gray scale) between the IDPH and the Mpipi models, considering only those interactions of residue *i* with residue *j* that satisfy *j* > *i* + 3, where only aromatic (orange), cationic (blue), negatively charged (red), and histidine (cyan) residues are plotted. The bottom right trigonal highlights the probability of cation−π and π–π interactions, both inclusive and exclusive of histidine, illustrating the involvement of histidine in the short-range interactions of Histatin 5. (B) Representation of three conformations of Histatin 5 simulated using the IDPH model that are stabilized by histidine cation−π (shown in green) and π–π (shown in magenta) interactions. (C) Changes to the map of inter-residue contacts within Histatin 5 (variant 6) as calculated by the IDPH (pH < 7) model referenced against the Mpipi model. The negative values of the color bar indicate loss of interactions when using the IDPH model at low pH. (D) The average energetic strength of short-range His–His, His–Arg, and His–Phe/Tyr interactions as simulated by the IDPH model (both low and high pH cases) and the Mpipi model. The standard deviation from the mean energy is plotted.

To understand the predominant interactions at low pH, we performed a similar comparison for the contact probabilities of Hst5 by subtracting those simulated using the Mpipi model from those obtained from the simulations using the IDPH model, with all His residues represented by His^+^ (see Figure 4C). The contact regions in which we found differences between the IDPH and Mpipi simulations were the same at low pH as at high pH (Figure 4A), but with an opposite trend (negative values) observed at low pH, such that the Mpipi model populates these contact probability regions more frequently than does the IDPH model. This difference likely reflects an interplay between two competing interactions: the stronger contribution of electrostatic repulsions between positively charged His residues but also stronger attraction due to cation−π interactions between His^+^ and aromatic residues. However, the magnitude of the difference between the contact maps at low pH is much smaller than at high pH (Figure 4A vs 4C). This suggests that the Mpipi and IDPH models behave similarly at low pH, despite these different interactions.

To further explore the energetic contributions of these interactions, we compared the mean energy terms for His–His, His–Arg, and His–Phe/Tyr interactions in both the IDPH model (at low or high pH) and the Mpipi model (Figure 4D). Interestingly, at low pH the mean His–Arg and His–His short-range energies obtained from the Mpipi model resembled those obtained from the IDPH model. These two models also agree that at low pH, Hst5 is dominated by cation−π interactions between His^+^ and Phe or Tyr. The energetics of His interactions in Hst5 differ when simulated at high pH compared with low pH, with calculations at high pH reflecting a high frequency of His–His and His–Arg pairwise interactions (π–π and cation−π, respectively) that are effective for the His^0^ state. The mean energies of His–Phe/Tyr contributions at higher pH values are similar to those obtained from the Mpipi model. This suggests that, whereas the electrostatic charge on His is averaged in the Mpipi model (and then adjusted to +0.375), the short-range contributions are not averaged but rather reflect a mix of low and high pH conditions.

*Different Histidine Interactions Govern the Response of CPEB4 and CPEB4Δ to pH*. The ability of the IDPH model to distinguish between the pairwise interactions of the two protonation states of His enabled us to study the effect of the pH on the functional dynamics of larger His-rich IDPs. Of particular interest is the neuronal CPEB4 protein, whose long 448-residue IDR was experimentally shown^3^ to exhibit strong interactions between its 9His-cluster (positions 229–252) and an 8-residue region termed microexon4 (me4, namely, the ARTYGRRR sequence at positions 403–410) at high pH. Deleting the me4 region produces the CPEB4Δ IDR variant (which has a 440-residue N-terminal domain). Employing the Mpipi model, we found similar mean Rg values of ∼49 Å for both the CPEB4 IDR and the CPEB4Δ IDR (Supporting Information Table S2), indicating no effect of me4 on overall protein dimensions. A similar trend was observed with the Control IDPH model, which yielded mean Rg values of 47 Å for CPEB4 IDR and 48 Å for CPEB4Δ IDR at high pH.

Different behavior was observed when the IDPH model was employed to assess His interactions within CPEB4 IDR and CPEB4Δ IDR under conditions of both high and low pH (Figure 5A,B). Interestingly, at high pH the Rg values of CPEB4 IDR and CPEB4Δ IDR differed (43 Å vs 46 Å, respectively). This result is surprising as it indicates that a mere eight-residue deletion results in expansion of the protein. To understand the interactions that govern these dimensions, we performed further analysis focusing on interactions between cationic (Lys, Arg, or His^+^) and aromatic (Phe, Tyr, Trp, or His^0^) amino acids. At high pH, the IDPH model simulated frequent interactions between the me4 and 9His-cluster regions (Figure 5A), thereby suggesting the presence of attractive His^0^–Arg cation−π interactions between these regions (Figure 5E). Frequent interactions between the me4 region and the 9His-cluster within the IDR of the CPEB4 monomer align with NMR experiments indicating that, even under denaturation conditions (4 M urea), these monomeric CPEB4 regions are not very solvent-exposed.^3^ These experimentally predicted interactions were absent when repeating this analysis with the Mpipi and Control IDPH models (see Supporting Information Figure S10), thus suggesting that His^0^–Arg cation−π interactions, which are not considered in these models, are important drivers of close interactions between these me4 and H-cluster regions.

**Figure 5:**
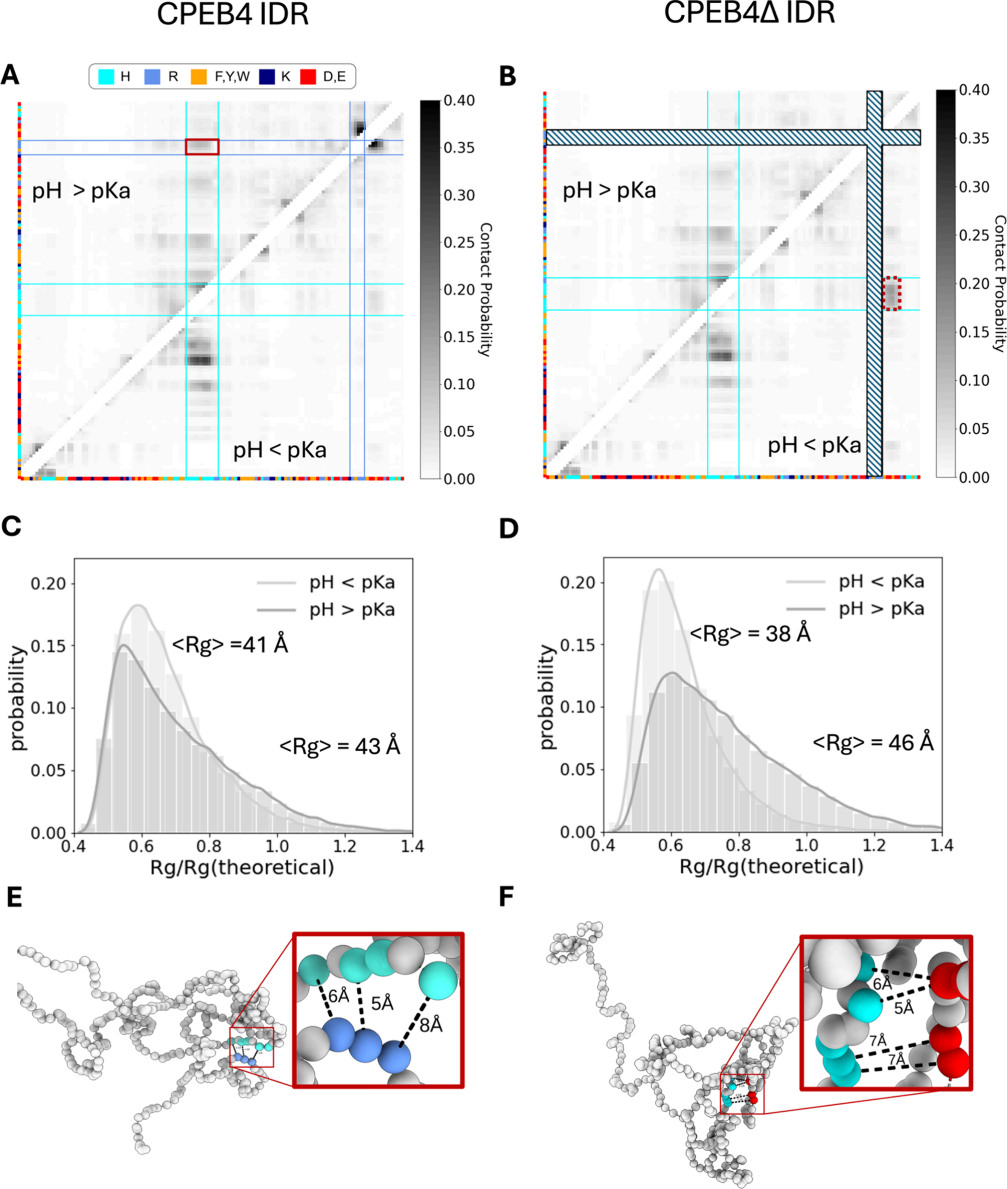
Conformational analysis of CPEB4 and CPEB4Δ disordered regions at low and high pH. (A) Contact map for CPEB4 IDR under conditions of low and high pH (lower and upper diagonals, respectively). Given the length of the CPEB4 IDR, the maps show the probability of contact only between aromatic, acidic, cationic, and histidine residues along the sequence (gray scale bar). The interacting region of the His-cluster (marked by light blue) and the me4 region (marked by dark blue) is highlighted by a red box. (B) Contact map for CPEB4Δ IDR under conditions of low and high pH (lower and upper diagonals, respectively), where the absence of the mex4 region is shown as striped lines. Interactions between the His-cluster and a region of negative residues in the vicinity of the position of the me4 region is indicated by a red dashed box. (C) Probability distribution of the simulated Rg values of the CPEB4 IDR at low and high pH values. To highlight the compaction of CPEB4 IDR, the Rg is normalized against the theoretical Rg value as estimated using the Flory scaling law for an IDP according to the relation 2.54·*N*^0.52^, where *N* is the length (measured as the number of amino acid residues) of the IDR of CPEB4 (*N* = 448). (D) As per panel C but for CPEB4Δ IDR (*N* = 440). (E) Representation of a CPEB4 IDR conformation stabilized by interactions between the His-cluster and the me4 region. (F) Representation of the conformation of CPEB4Δ IDR stabilized by interactions between the His-cluster and the negative residues in the vicinity of the me4 region.

At low pH, however, the IDPH shows that these regions rarely interacted because of electrostatic repulsion between His^+^ and the four Arg residues in me4. Instead, the His-cluster interacted more frequently with both aromatic amino acids (via His^+^ cation−π interactions) and negatively charged amino acids (via charge–charge interactions) that lie closer to the N-terminal. Despite these pH-dependent changes in amino acid residue interactions, the average size (Rg) of CPEB4 IDR remained relatively similar, with a slight extension at higher pH (41 Å with His^+^ vs 43 Å with His^0^, a difference of ∼5%) (Figure 5C). In contrast to CPEB4 IDR, the Rg of CPEB4Δ IDR was found to be more affected by pH, increasing more significantly from 38 to 46 Å at higher pH (an increase of ∼20%) (Figure 5D). These observations suggest that the absence of me4 leads to opposing effects on protein dimensions, with compaction (by ∼3 Å) observed at low pH and expansion (by ∼3 Å) at higher pH, compared with the WT CPEB4 IDR. The smaller effect observed in the presence of me4 is likely due to the presence of multiple cooperative His^0^–Arg attractive interactions involving me4 (Figure 5E).

Indeed, for the shorter variant CPEB4Δ IDR (see Figure 5B) at high pH, we observed infrequent interactions (compared with those of WT CPEB4 IDR) between the 9H-cluster and the 403–410 amino acid region (which corresponds to the me4 region in CPEB4 IDR but not in CPEB4Δ IDR, which lacks the me4 region). Conversely, at low pH, the positively charged 9His-cluster interacted more frequently with a negatively charged region in the former position of the spliced me4 (compared with the WT CPEB4 IDR at low pH) (see Figure 5F). This over-representation of contacts in the spliced variant suggests that, although this region exists in the WT protein, me4 primarily blocks this interaction at low pH via electrostatic repulsion. This is further supported by a calculated Rg of 38 Å for the spliced CPEB4Δ IDR variant (Figure 5D), indicating a more compact structure compared to the WT protein.

We note that, regardless of the charge on His, the predicted Rg values for CPEB4 variants were significantly smaller than theoretically expected for an IDP of the same size^31^ (the calculated peak probability occurs at Rg/Rg(theoretical) ≈ 0.6, Figure 5C,D), thus implying that CPEB4 variants adopt a more compact structure under both pH scenarios due to cooperative stabilizing interactions. Our results suggest that the compact nature of CPEB4 IDR variants underscores the importance of considering different His interactions, such as His^0^–His^0^ and His^0^–Arg interactions, in the CG modeling of His-rich disordered proteins.

In conclusion, computational CG modeling of IDPs often simplifies His interactions, focusing primarily on electrostatic interactions and neglecting the crucial role of short-range interactions such as cation−π and π–π interactions. Here, we incorporated these interactions into an upgraded CG model, called the IDPH model, which includes two independent protonation states of His (*i*.*e*., His^0^ and His^+^), and studied their contributions to the conformational ensembles of IDPs under two extreme scenarios in which all His residues were either neutral (pH > p*K*_a_) or positively charged (pH < p*K*_a_).

The IDPH model produces better agreement with the experimentally determined dimensions of different His-rich variants of the Hst5 IDP than earlier models, including the Mpipi. Under high pH conditions, the conformations of Hst5 are stabilized primarily by His interactions that are completely invisible to the previous CG models. The conformations of the IDPs are stabilized by the interactions of His with aromatic and basic residues via π–π and cation−π interactions, respectively, which are also supplemented by neighboring π–π and cation−π interactions involving other residues (e.g., Phe–Phe and Phe–Arg) that have comparable or higher strengths than π–π interactions involving His and that are already adequately represented in earlier models. The conformations of Hst5 are quite different under conditions of low pH when, according to the IDPH model, His^+^ participates in electrostatic interactions with Glu or Asp and engages in cation−π interactions with aromatic residues. The conformational ensemble involving His^+^ sampled by the IDPH model is broadly consistent with that sampled by the Mpipi model. However, at higher pH values, the IDPH better captures experimental Rg due to the improved energetics of some cation−π and π–π interactions (particularly, His^0^–His^0^ and His^0^–Arg) compared with the Mpipi model.

Applying the IDPH model to the CPEB4 IDR provided insights into the effect of His on pH-dependent IDP conformations. We observed that the CPEB4 IDR maintains a relatively constant size under low and high pH conditions, despite changes in intramolecular interactions. Investigation of this shift in interactions showed that, under high pH conditions, His^0^–Arg interactions involve the me4 region, whereas under lower pH conditions, the conformation of CPEB4 IDR is influenced by electrostatic attractions between His residues and negatively charged regions and aromatic residues. The shorter CPEB4Δ IDR variant that lacks the me4 region presents an interesting contrast to the WT CPEB4 IDR. In CPEB4Δ IDR, the absence of His^0^–Arg interactions at high pH levels promotes expansion, whereas electrostatic attractions dominate at low pH, leading to compaction. This demonstrates how even minor sequence alterations can dramatically alter the interplay between His interactions and pH, leading to opposing effects on IDP conformations.

In summary, this study emphasizes the critical need for accurate modeling of His interactions to understand the structure and function of His-rich disordered proteins. Our IDPH model provides a valuable tool for studying His-rich proteins under low and high pH conditions. There remains a need for a model that includes a fluctuating protonation state of His with a dynamic mixture of His^0^ and His^+^ depending on the local environment and exact pH, particularly for pH ≈ p*K*_a_. Such a subtle model for studying the conformational dynamics of IDPs will be addressed in future work.

## Methods

*Implementing Histidine Interactions in CG Molecular Dynamics Simulations of Disordered Proteins*. The various pairwise interactions in which His can engage were implemented in a CG molecular dynamics model in which each amino acid is represented by a spherical bead centered on the residue’s *C*_α_ position, as is often done in the study of IDPs.^23−25,32,33^ The potential energy function satisfies
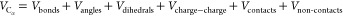
The bonded and angular contributions are
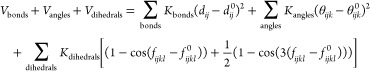
where *d*_*ij*_ is the distance (Å) between bonded beads *i* and *j*; θ_*ijk*_ is the angle (radians) between three adjacent beads *i*, *j*, and *k*; and *f*_*ijkl*_ is the dihedral angle between residues *i*, *j*, *k* and residue *l*.

The conditions , and  apply except when a proline residue is involved, in which case dihedral angles with force constants (*K*_*ijkl*_) and equilibrium angles *f*_ijkl_^0^ are introduced, following another work.^34^

The intramolecular short-range contacts within the IDP are modeled by the Lennard-Jones (LJ) 10-12 potential as follows:
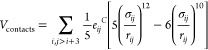
where the strength of a pairwise interaction between residue *i* and its partner *j* is defined by *e*_*ij*_^*C*^. Several CG models for IDPs (such as the hydrophobicity scale (HPS), Kim-Hummer (KH),^25^ Urry-HPS,^35^ and FB hydrophobicity (FB-HPS)^36^ models) define the values of *e*_*ij*_^*C*^ solely on the basis of the hydrophobicity of the interacting residues, whereas the IDPH model scales *e*_*ij*_^*C*^ according to both the hydrophobicity of the interacting residues and the strength of the π–π and cation−π interactions, as does the multiscale π–π (Mpipi)^23^ CG model. The distance σ_*ij*_ dictates the optimal distance for each pairwise interaction. For further details, refer to the Supporting Information.

To introduce pH-dependent His interactions into IDPs, we developed the IDPH CG model, which distinguishes between His^0^ and His^+^ states. The IDPH model thus includes long-range interactions (*i*.*e*., electrostatic interactions) and short-range interactions (*i*.*e*., both π–π and cation−π interactions) undertaken by His with relevant partner residues. Given the expected significance of cation−π and π–π interactions for His, the IDPH model is constructed by adopting the strength of non-His pairwise interactions from the Mpipi model, which was parametrized by acknowledging cation−π and π–π interactions for aromatic residues. The Mpipi model was shown to capture the experimental radii of gyration (Rg) values of 17 different IDPs.^23^ Yet, in the Mpipi model, His has a constant +0.375 charge; thus, the model cannot assess the effect of pH on histidine’s interactions. Furthermore, the strength of His^0^–His^0^ interactions in this model is about 14 times weaker than the Phe-His, despite their strength being comparable based on QM calculations.^2^ This is due to the Mpipi averaging over all His–His combinations observed as His^0^–His^0^, His^0^–His^+^, and repulsive His^+^–His^+^. Similarly, the His^0^–Arg interaction is underrepresented by the Mpipi, which models them as being less than a quarter of the strength of Phe–Arg interactions, inconsistent with our observation of comparable π–π and cation−π interaction strengths for neutral His.^2^

To include the His^0^–His^0^ and His^0^–Arg interactions in the IDPH model, we treated His^0^ and His^+^ as separate amino acids with only the latter participating in electrostatic interactions. We further optimized the strengths of short-range interactions involving His^0^ and His^+^, based on our quantum mechanical average binding energies calculation (reported elsewhere^2^). These were linearly rescaled to match the commonly discussed interacting pairs depending on the partner with which His interacts. The strength of His^0^–His^0^ interactions was calibrated based on other π–π pairs, specifically, Phe–Phe, Phe–Tyr, Phe–Trp, Tyr–Tyr, Tyr–Trp, and Trp–Trp, as shown in Supporting Information Figure S2. We find our quantum mechanical data to be strongly correlated (*R*^2^ ≈ 0.99) with the current strengths of these π–π pairs, with only His^0^–His^0^ interactions considerably underestimated in the Mpipi model. Similarly, the strength of His^0^–Arg contacts (Supporting Information Figure S2) was calibrated based on the strengths of the cation−π pairs Arg–Phe, Arg–Tyr, and Arg–Trp (*R*^2^ ≈ 0.95). To avoid confusion between the representation of His in Mpipi (as His^+^, modeled with a charge of +0.375, regardless of the pH) and His^+^ in IDPH (modeled with a charge of +1, for pH < p*K*_a_), we refer to IDPH conditions as either low pH (pH < 7; *i*.*e*., His modeled as His^+^) or high pH (pH > 7; *i*.*e*., His modeled as His^0^).

For charge–charge contributions, we used electrostatic interactions screened by the Debye–Hückle potential:
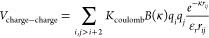
where *q*_*i*_ and *q*_*j*_ are the electrostatic charges on beads *i* and *j*, respectively, and *r*_*ij*_ is the distance between this pair of residues (Å), *K*_coulomb_ = 332 kcal/mol, ε_*r*_ = 80 is the dielectric constant of the solvent, κ is the reciprocal of the Debye screening length, which is proportional to the root of the ionic strength, and *B*(κ) is the salt-dependent coefficient.

*Details of the Simulations*. Langevin dynamics coarse-grained simulations (Velocity Verlet algorithm) were performed employing an IDP model^34,37^ using an in-house code, where all residue pairs that satisfy |*i* – *j*| > 3 have additional short-range energetic contribution based on their contact strengths (*i*.*e*., the value of *e*_*ij*_^*C*^). Simulations were run for 2 × 10^8^ molecular dynamics (MD) steps (equivalent to a 10 μs time scale where a step is 50 fs) with an output frequency of 1–1000 frames saved. Ionic salt equivalent to ∼150 mM was used, as in this condition our implementation of Mpipi reproduced the computational dimensions reported for 17 proteins studied with the Mpipi model.^23^ For Histatin variants, a 120 mM salt concentration was used, following the salt concentration used experimentally,^29^ along with a temperature of 0.45 reduced units and a bead mass of 1 reduced units (all energies are in *K*_B_*T* units with *K*_B_ being the Boltzmann constant; for more details refer to the following MD code repository: https://github.com/rivkacal/pH-dependentCG), which corresponds approximately to room temperature.^38^ For each system, five simulation replicates were conducted (*i*.*e*., simulations with different initial velocities). For CPEB4 variants, considering their larger dimensions, 10 repetitions were performed.

Rg values are reported as the arithmetic average of all included timesteps (∼5 × 10^6^ equilibration time steps were removed from the analysis) among all five repetitions. The error was estimated as the standard deviation of the Rg value from 5 (or 10) repetitions.

The mean squared error (MSE) for *n* samples with computational *Rg*_*n*_ compared with experimentally reported *Rg*_exp,*n*_ was calculated using
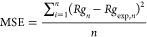
The χ^2^ error is as follows:
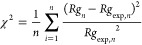
Contact maps were acquired by using a cutoff threshold of 10 Å to define a contact (thus including all Lennard-Jones contributions and the majority of electrostatic interactions).

## Supporting Information Available

Modifications in coarse-grained modeling of short-range interactions; calibration of his interaction strengths in IDPH; Pperformance of Mpipi and IDPH for a set of 18 His-inclusive sequences; correlation between IDPH and Mpipi even for similar Rg of sfAFP suggests differences in interactions; effect of his’ content on disordered peptide dimensions; performance of different CG models; control IDPH model: importance of H^0^ short-range interactions; contribution of His^0^ modeled cation−π interactions within IDPH; impact of single his residue protonation; effect of pH on monomeric CPEB4 dimensions and interactions (PDF)
