# Supplementary material for: A pH-Dependent Coarse-Grained Model for Disordered Proteins: Histidine Interactions Modulate Conformational Ensembles
Source: J Phys Chem Lett. 2024 Sep 9;15(37):9419–30. doi: 10.1021/acs.jpclett.4c02314 (PMC11417990; doi:10.1021/acs.jpclett.4c02314)
Supplement: Supplementary file 1 — jz4c02314_si_001.pdf [file jz4c02314_si_001.pdf]

Supporting Information

A pH-Dependent Coarse-Grained Model for Disordered Proteins: Histidine Interactions Modulate Conformational Ensembles

Rivka Calinsky and Yaakov Levy*

Department of Chemical and Structural Biology

Weizmann Institute of Science

Rehovot, 76100, Israel

*Corresponding author: Yaakov Levy, Department of Chemical and Structural Biology, Weizmann Institute of Science, Rehovot, 76100, Israel; email: Koby.Levy@weizmann.ac.il; Tel: 972-8-9344587

Modifications in Coarse-Grained Modeling of Short-Range Interactions

KH Model

We note that our implementation of KH model slightly differs from the original[1]. While the original model implies some repulsive contributions between specific amino-acids pairs (i.e., ${\varepsilon_{ij}}^C < 0$), we chose instead to assign a value of 0; Thus, such pairs interactions follow only excluded-volume contributions:

$$V_{\text{non-contacts}} = \sum_{i,j \in non-contacts} K_{repulsions} \left(\frac{\sigma_{ij}}{r_{ij}}\right)^{12}$$

Where $K_{repulsions} = 1$kcal/mol.

Mpipi Model

We note key differences between our implementation of IDPH and Mpipi model and the reported model[2]. For the Mpipi model the electrostatic charge of charged amino acids was factored by 0.75. For example, Arg and Lys are assigned qi,j of +0.75, Histidine +0.375 and Asp+Glu share -0.75. Our implementation of Mpipi indeed includes the +0.75 factor, however this factor is not used for the IDPH model. To include the effect of this factor we repeated the calculations of this work, using the terminology of 'Mpipi Fullcharge' model. To include the effect of correct His charge representation (i.e., neutral charge at pH > pKa), we designed a control, "Control Mpipi model". Similarly, to investigate the effect of pure electrostatic charge of His in IDPH, we refer to "Control IDPH model", in which the energetic terms of His^0-His^0 and His^0-Arg were turned off compared to IDPH.

While Mpipi originally employ a different potential for specific amino acids pairs (as isoleucine), our implementation does not include for these values. Instead, we used only a single unified potential to represent all the contacts. These should not affect our insights regarding Histatin 5 variants as they do not include Ile residues. Furthermore, the CG models implemented in this work (HPS, KH, Urry and FB) employ the Lennard-Jones like potential (refer to the Methods section in the main text) to represent contacts between sidechains of interacting amino-acids. However, the Mpipi model originally employ the Wang-Frenkel potential (WF) obeying:

$$V_{LJ}^{WF} \propto {\varepsilon_{ij}}^C \left[\left(\frac{\sigma_{ij}}{r_{ij}}\right)^4 - 1\right]\left[\left(\frac{3\sigma_{ij}}{r_{ij}}\right)^4 - 1\right]^2$$

To consistently use the same Lennard-Jones potential also for the Mpipi model, the σ_{ij} velues reported using the WF potential (see Fig. S1.A), were factored by ~1.105 factor to align the LJ potential (employing the newer σ_{ij}) with the minimum position of the WF potential (see Fig. S1B).

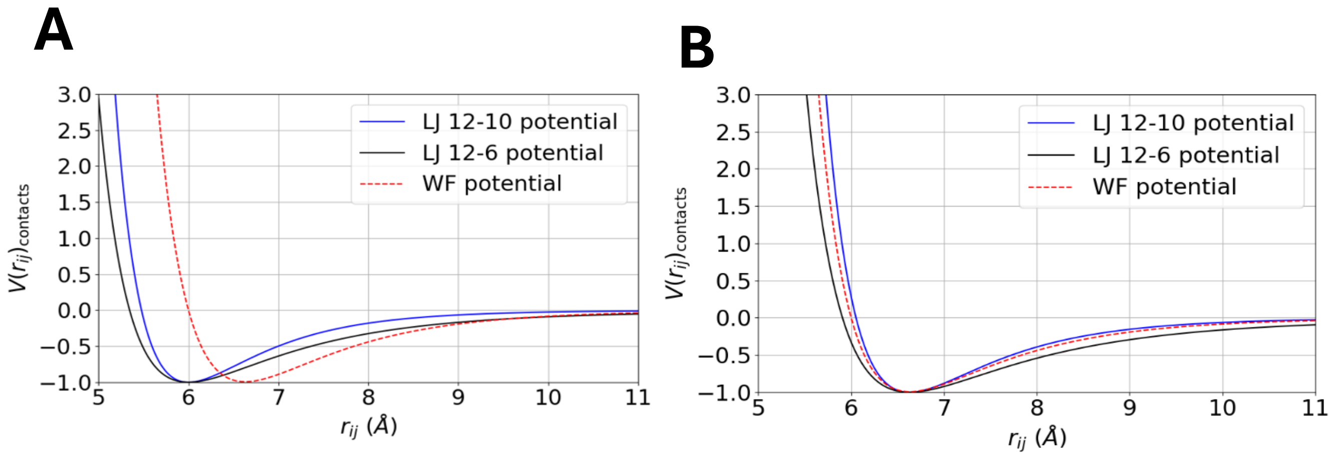

Figure S1: Comparison of different short-range potentials. (A) The short-range potentials are represented by choosing $\sigma_{ij} = 6$Å. The "LJ 12-10" and "LJ 12-6" potentials' minima overlap at $\sigma_{ij} = 6$Å. Conversely, the WF potential intersects $V_{LJ}^{WF} = 0$ at $\sigma_{ij} = 6$Å.
(B) LJ potentials shifted to the position of WF minima using a ~1.105 factor, as employed in our model (Mpipi and IDPH).

Calibration of His Interaction Strengths in IDPH

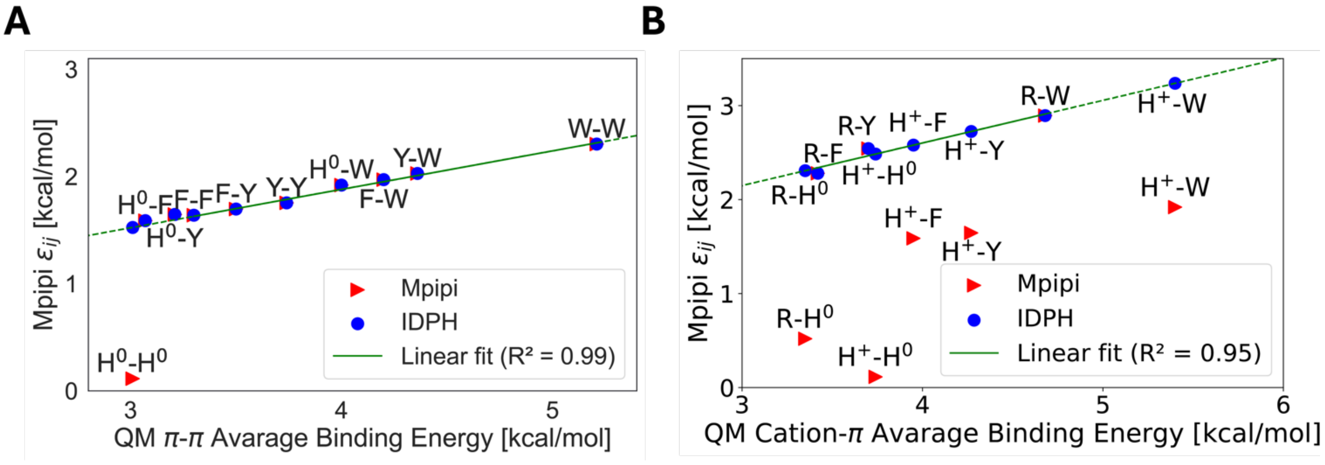

Figure S2: Calibration Histidine short-range interactions in IDPH. A). The strength of pairwise interaction between residue i and its partner j, $\varepsilon_{ij}{}^{C}$ (see Methods), for aromatic-aromatic (π-π) pairs as a function of the QM average energies of π- π interactions for the same pairs[3] . The strength of Phe-Phe, Phe-Tyr, Tyr-Tyr, His^0-Tyr, and His^0-Trp were kept the same in IDPH model as Mpipi. Only the strength of His^0- His^0 was refined to match with the calibration line for the other interactions. **B).** Calibration of cation- π interacting pairs strengths. Arg- His^0, His$^+$- His^0, His$^+$-Phe, His$^+$-Tyr, His$^+$-Trp were refined to match with the calibration line of Arginine as a cation (Arg-Phe, Arg-Tyr, and Arg-Trp). Lys' interactions were kept as in the Mpipi model following their observed low abundance both in the Mpipi model [2] and in our work [3].

Performance of Mpipi and IDPH for a set of 18 His-inclusive sequences

Table S1: Rg values calculated using Mpipi and IDPH model compared to the SAXS determined experimentally reported values. Proteins whose computationally calculated values match the experimental values within the error are colored in green for convenience. Experimental Rg values including errors (when reported) can be found in the following works [2,4–10].

Model	Frag	Rg (Å)	s.d (Å)	Experimental Rg (Å)	Experimental error (Å)
IDPH	GHRICD	66.4	0.8	62.2	5.9
IDPH	CORNID	51.9	0.5	48.4	1.1
IDPH	AB40	18.0	0.0	10.0	1
IDPH	AB42	18.6	0.0	16.0	1
IDPH	AtPARCL	34.2	0.5	34.7	/
IDPH (pH < 7)	GRDBD94	30.5	0.1	25.0	0.5
IDPH	sfAFP	29.9	0.2	24.5	0.12
IDPH	TtASR1	37.2	0.2	35.5	0.3
IDPH (pH < 7)	Tat	30.5	0.1	34.4	0.3
IDPH	alphaSynuclein	39.8	0.4	33.1	0.3
IDPH	ACTR	27.5	0.1	25.1	/
IDPH	Ash1	31.3	0.1	28.5	3.4
IDPH	hNHE1cdt	36.5	0.2	36.3	/
IDPH	K18	38.3	0.2	38.0	0.3
IDPH	K25	40.8	0.4	41.0	0.2
IDPH	SH4UD	26.1	0.1	28.2	0.4
IDPH	Hst5 (seq 5)	12.4	0.02	13.5	0.01
IDPH	Hst5chained (seq 7)	17.8	0.1	18.5	0.1
IDPH	Hst5RAN (seq 6)	12.6	0.03	11.9	0.06
IDPH	Hst5DZM (seq 4)	12.9	0.06	12.1	0.07
IDPH	HstZM (seq 3)	13.0	0.03	12.8	0.05
IDPH	Hst52H (seq 2)	13.2	0.03	14.1	0.04
IDPH	Hst50 (seq 1)	13.5	0.03	13.9	0.13
IDPH one charged	Hst5chained (seq 7)	18.5	0.09	18.5	0.1
Mpipi	GHRICD	65.1	0.6	62.2	5.9
Mpipi	CORNID	54.7	0.7	48.4	1.1
Mpipi	AB40	18.1	0.1	10.0	1
Mpipi	AB42	18.7	0.1	16.0	1
Mpipi	AtPARCL	38.1	0.3	34.7	/
Mpipi	GRDBD94	29.9	0.1	25.0	0.5
Mpipi	sfAFP	30.3	0.1	24.5	0.12
Mpipi	TtASR1	36.4	0.3	35.5	0.3
Mpipi	Tat	29.0	0.1	34.4	0.3
Mpipi	alphaSynuclein	40.5	0.3	33.1	0.3
Mpipi	ACTR	27.1	0.1	25.1	/
Mpipi	Ash1	30.8	0.1	28.5	3.4
Mpipi	hNHE1cdt	37.6	0.2	36.3	/
Mpipi	K18	38.3	0.2	38.0	0.3
Mpipi	K25	45.8	0.2	41.0	0.2
Mpipi	SH4UD	26.7	0.1	28.2	0.4
Mpipi	Hst5	14.3	0.02	13.5	0.01
Mpipi	Hst5chained	23.3	0.04	18.5	0.1
Mpipi	Hst5RAN	14.3	0.04	11.9	0.06
Mpipi	Hst5DZM	13.7	0.03	12.1	0.07
Mpipi	HstZM	13.5	0.02	12.8	0.05
Mpipi	Hst52H	13.4	0.03	14.1	0.04
Mpipi	Hst50	13.3	0.01	13.9	0.13

Correlation between IDPH and Mpipi even for similar Rg of sfAFP suggests differences in interactions

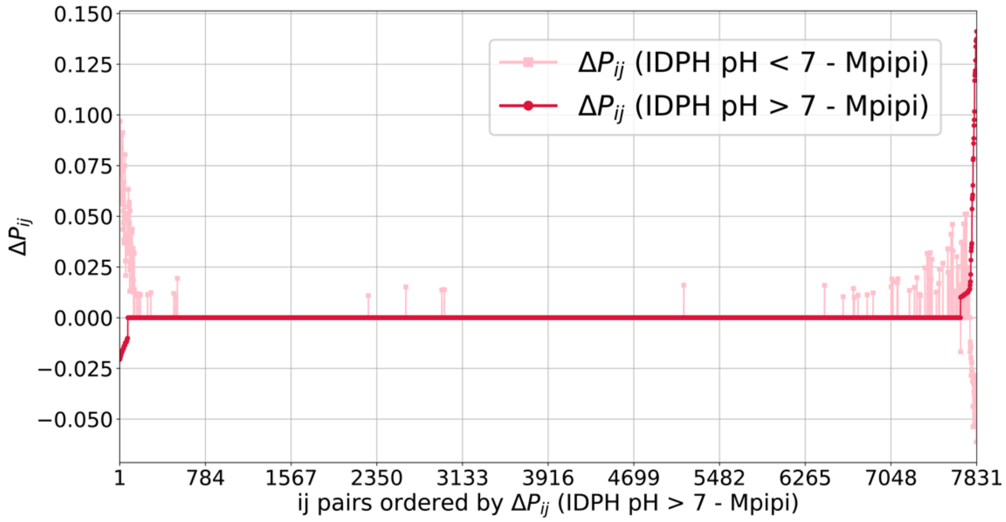

Figure S3: The difference in probability for a contact between residue i and residue j (ΔPij) as predicted by IDPH against Mpip. High pH is colored in crimson and low pH in pink. These Pij values were sorted by the magnitude of ΔPij for the higher pH difference case for convince. Values smaller than 0.01 (absolute values) were nullified to highlight cases where the changes in probability are more significant.

The Effect of His' Content on Disordered Peptide Dimensions

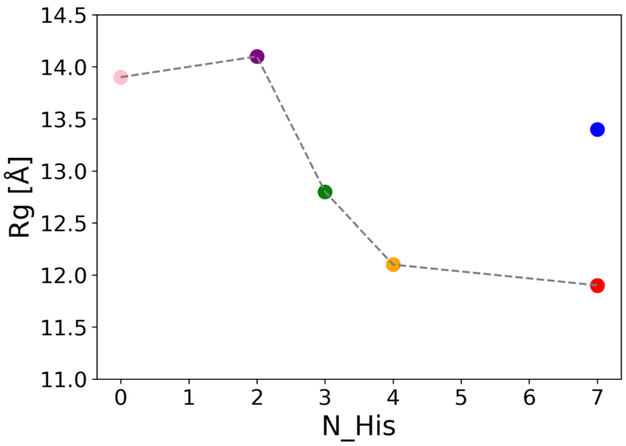

Figure S4: Experimental Rg of Histatin's variants as a function of His content. Values were taken from the following work [11].

Performance of Different CG Models

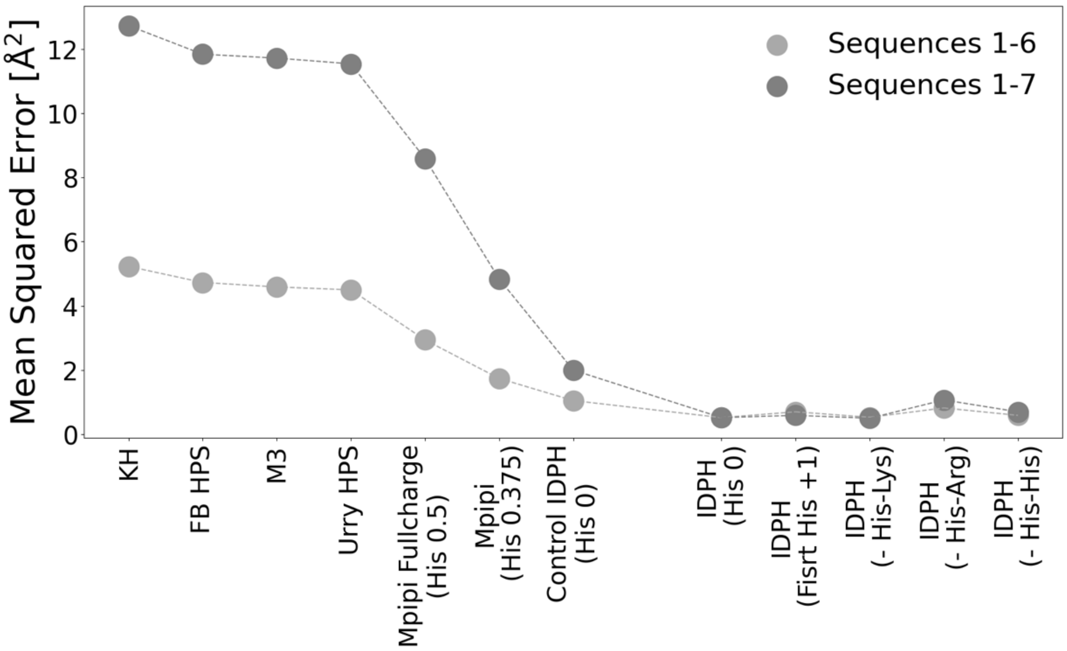

Figure S5: The MSE values for the correlations between the computational Rg the variants of Histatin5 simulated using various models to their experimental Rg. The correlation is quantified by the MSE value for variants 1-7 (dark grey circles) or variants 1-6 (light grey circles). The electrostatic charge of His is mentioned in brackets for comparison of different models, otherwise it is +0.5.

Control IDPH Model: Importance of H^0 Short-Range Interactions

To validate the importance of short-range interactions of His, we checked whether a simple implementation of only assignment of the physical charge state of His within Mpipi (i.e. neutral charge instead 0.375) is enough to explain the experimentally observed dimensions, as shown in Fig.S6 below.

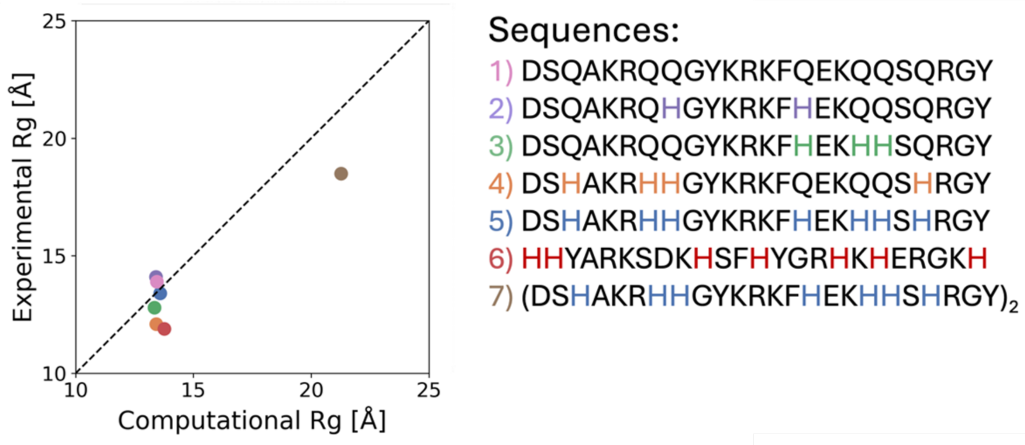

Figure S6: The role of H^0 short-range interactions. The results are plotted employing the Control IDPH model as discussed in the main text and the supporting section S1.

The contribution of His^0 modeled cation- π interactions within IDPH

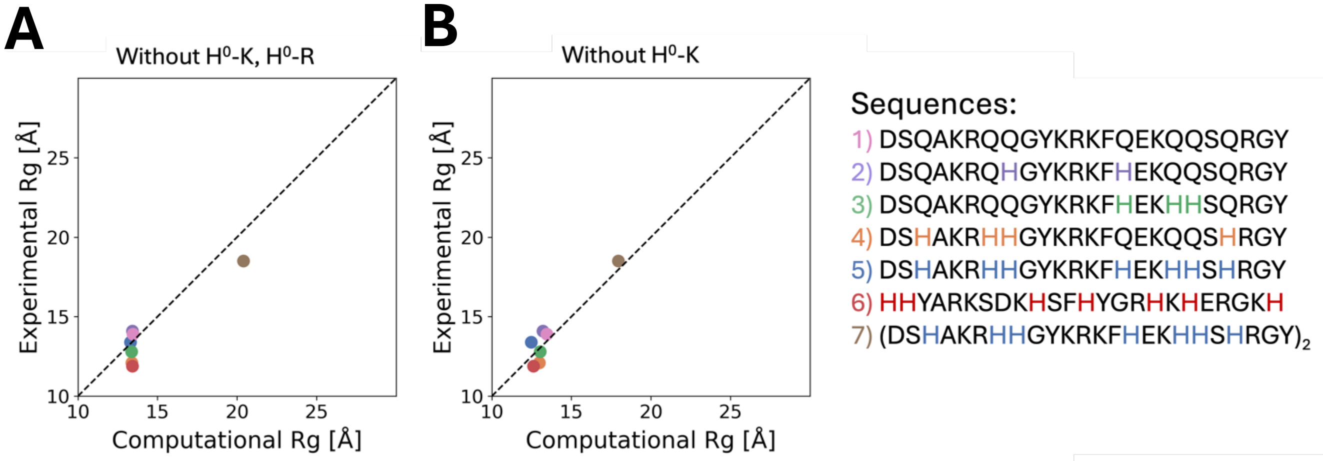

Figure S7: The role of H^0 cation- π interactions. (A) IDPH model (pH > 8) excluding the contributions of H^0 cation- π (both H^0-K and H^0-R). (B) Excluding only H^0-K.

Impact of Single His residue Protonation

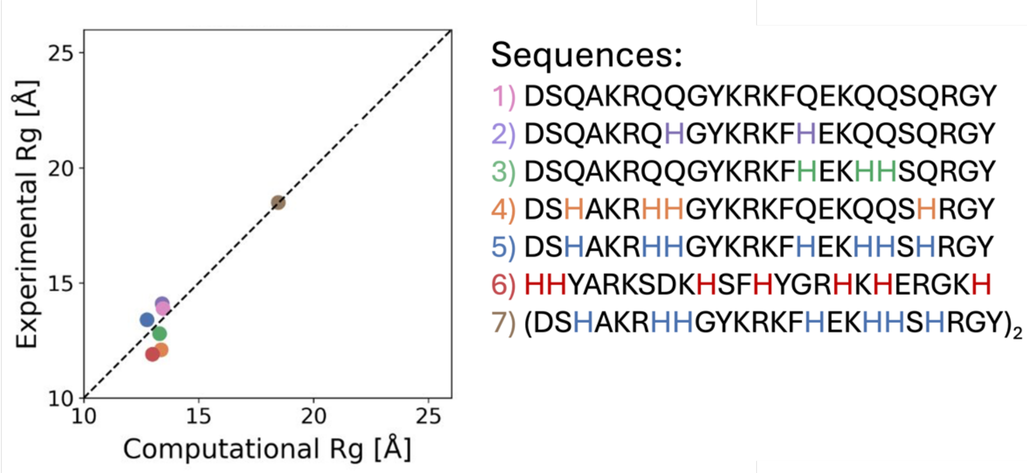

Figure S8. The effect of a single H^+ residue. Performance of IDPH model where all His residues are assigned neutral charge except for the first His residue assigned as H^+.

Effect of pH on Monomeric CPEB4 Dimensions and Interactions

The dimension of the NTD CPEB4 monomer has been reported experimentally, from DLS liquid exclusion chromatography[12]. The reported 10.9 nm hydrodynamic diameter for a monomer at high pH corresponds to 55 Å hydrodynamic radius. While this seems to fit the theoretical 55 Å Rh for 448 using R_0=3.53 and ν=0.449 (where Rh~ R_0N^{ν}, see Ref[13]), observing Figure 1B in this reference suggests that for a 55 Å Rh dimensions, the experimental radii are overestimated by ~25% (calculated ~40 Å and experimental ~50 Å). The use of denaturants has been previously shown to overestimate the size of IDPs where the effect of low salt concentration dramatically decreases the dimensions due to electrostatic screening enabling compaction [14,15]. Therefore, we cannot compare our calculated Rh (refer to Rh methods assessment in Ref[16] where method #4: Nygaard -KR performs best for proteins ~4-5nm Rg) for CPEB4, which is ~43 Å at high pH conditions. Since Rg and Rh are correlated (see Fig.S9 below) the main text discussed Rg as a dimensional observable for comparison.

Table S2: The average Rg and Rh of CPEB4 and CPEB4Δ as a function of pH, employing different CG models.

Model	pH	His charge	variant	Rg (Å)	Rh (Å)
	/	0.375	CPEB4	48.7 ± 0.7	47.8 ± 0.4
Mpipi	/	0.375	CPEB4Δ	49.1 ± 0.8	48.1 ± 0.4
Mpipi	/	0.5	CPEB4	43.9 ± 0.5	45.0 ± 0.3
Fullcharge	/	0.5	CPEB4Δ	43.7 ± 0.7	44.7 ± 0.4
	< 7	1	CPEB4	44.0 ± 0.4	45.1 ± 0.2
	< 7	1	CPEB4Δ	40.8 ± 0.8	43.2 ± 0.4
	> 7	0	CPEB4	46.6 ± 0.5	46.2 ± 0.3
Control IDPH	> 7	0	CPEB4Δ	47.9 ± 1.0	46.8 ± 0.6
	< 7	1	CPEB4	40.9 ± 0.9	43.0 ± 0.6
	< 7	1	CPEB4Δ	38.4 ± 0.5	41.3 ± 0.4
	> 7	0	CPEB4	42.8 ± 0.8	43.0 ± 0.3
IDPH	> 7	0	CPEB4Δ	46.0 ± 1.2	44.9 ± 0.5

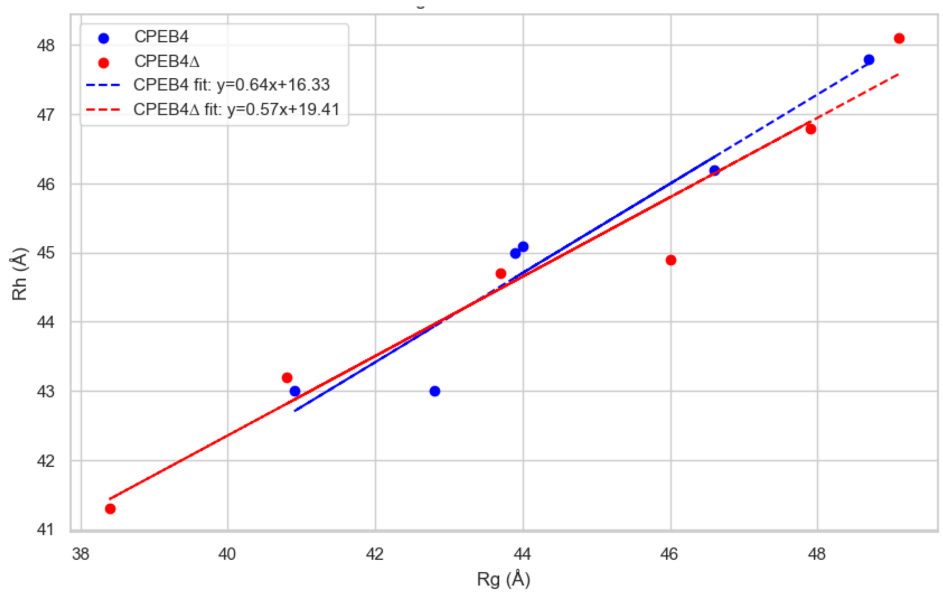

Fig. S9 correlation between Rh and Rg values for CPEB4 IDR variants.

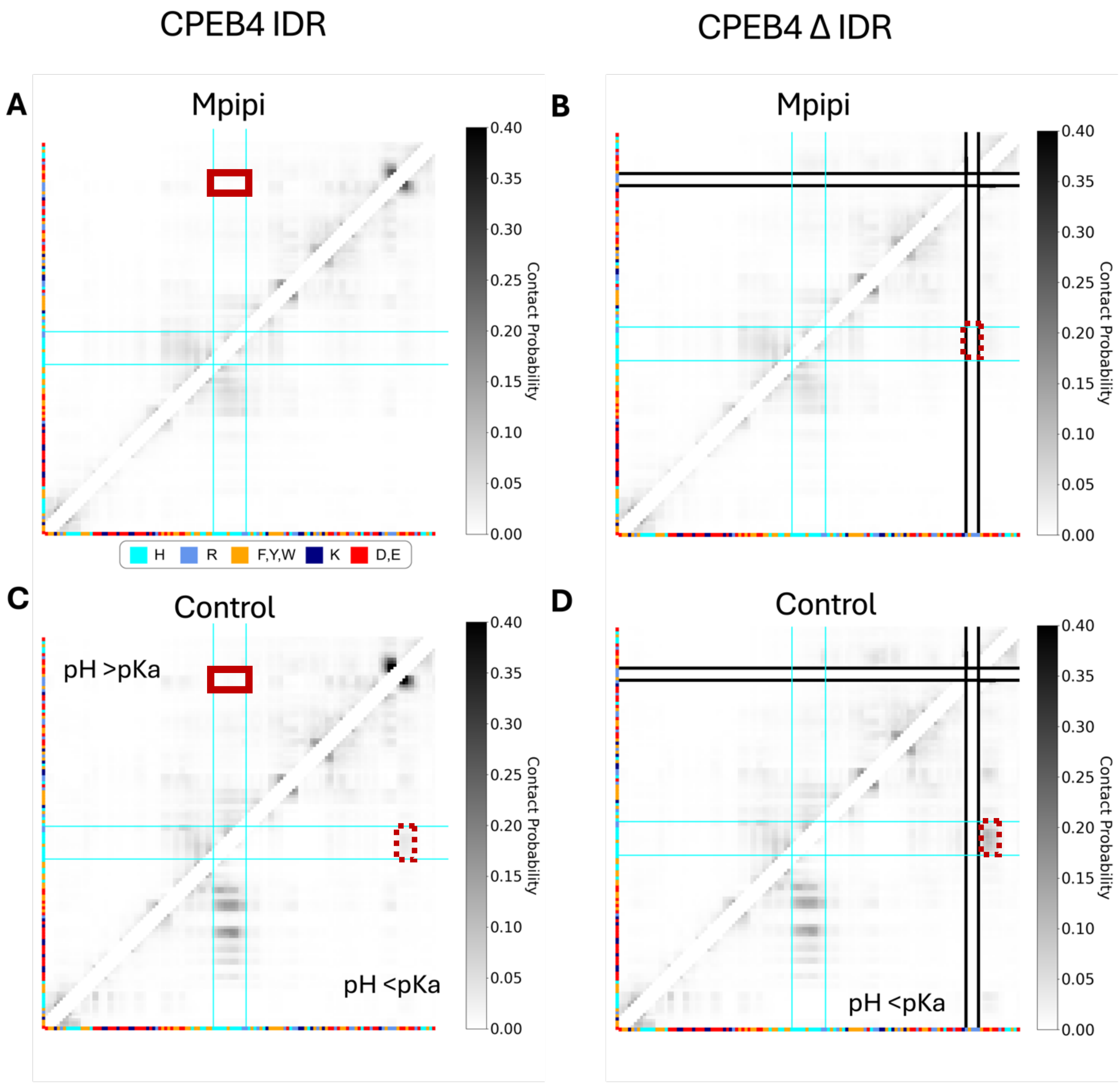

Fig. S10. Contact maps for IDR CPEB4 variants employing Mpipi with electrostatic +0.375 or without as Control display infrequent His-His or His-Arg interactions. For Control at pH < 7 a full charge is used for His instead +0.375 or 0.5. Otherwise, the Mpipi can be used as the control for low pH. At pH>7 Control IDPH is employed (i.e, His^0-His^0 and His^0-Arg were turned off).

References:

1. Dignon GL, Zheng W, Kim YC, Best RB, Mittal J. Sequence determinants of protein phase behavior from a coarse-grained model. *PLoS Comput Biol*. 2018;14(1). doi:10.1371/journal.pcbi.1005941
2. Joseph JA, Reinhardt A, Aguirre A, et al. Physics-driven coarse-grained model for biomolecular phase separation with near-quantitative accuracy. *Nat Comput Sci*. 2021;1(11):732-743. doi:10.1038/s43588-021-00155-3
3. Calinsky R, Levy Y. Histidine in Proteins: pH dependent interplay between π-π, cation-π, and CH-π interactions.
4. Mylonas E, Hascher A, Bernadó P, Blackledge M, Mandelkow E, Svergun DI. Domain conformation of tau protein studied by solution small-angle X-ray scattering. *Biochemistry*. 2008;47(39):10345-10353. doi:10.1021/bi800900d
5. Arbesú M, Maffei M, Cordeiro TN, et al. The Unique Domain Forms a Fuzzy Intramolecular Complex in Src Family Kinases. *Structure*. 2017;25(4):630-640.e4. doi:10.1016/j.str.2017.02.011
6. Araki K, Yagi N, Nakatani R, et al. A small-angle X-ray scattering study of alpha-synuclein from human red blood cells. *Sci Rep*. 2016;6. doi:10.1038/srep30473
7. Kjaergaard M, Nørholm AB, Hendus-Altenburger R, Pedersen SF, Poulsen FM, Kragelund BB. Temperature-dependent structural changes in intrinsically disordered proteins: Formation of α-helices or loss of polyproline II? *Protein Science*. 2010;19(8):1555-1564. doi:10.1002/pro.435
8. ash1.
9. Cragnell C, Staby L, Lenton S, Kragelund BB, Skepö M. Dynamical oligomerisation of histidine rich intrinsically disordered proteins is regulated through zinc-histidine interactions. *Biomolecules*. 2019;9(5). doi:10.3390/biom9050168
10. Fagerberg E, Månsson LK, Lenton S, Skepö M. The Effects of Chain Length on the Structural Properties of Intrinsically Disordered Proteins in Concentrated Solutions. *Journal of Physical Chemistry B*. 2020;124(52):11843-11853. doi:10.1021/acs.jpcb.0c09635

11. Cragnell C, Staby L, Lenton S, Kragelund BB, Skepö M. Dynamical oligomerisation of histidine rich intrinsically disordered proteins is regulated through zinc-histidine interactions. *Biomolecules*. 2019;9(5). doi:10.3390/biom9050168
12. Garcia-Cabau C, Bartomeu A, Balaceanu A, et al. Kinetic stabilization of translation-repression condensates by a neuron-specific microexon. doi:10.1101/2023.03.19.532587
13. Bernadó P, Blackledge M. A self-consistent description of the conformational behavior of chemically denatured proteins from NMR and small angle scattering. *Biophys J*. 2009;97(10):2839-2845. doi:10.1016/j.bpj.2009.08.044
14. Maity H, Baidya L, Reddy G. Salt-Induced Transitions in the Conformational Ensembles of Intrinsically Disordered Proteins. *Journal of Physical Chemistry B*. 2022;126(32):5959-5971. doi:10.1021/acs.jpcb.2c03476
15. Soranno A, Koenig I, Borgia MB, et al. Single-molecule spectroscopy reveals polymer effects of disordered proteins in crowded environments. *Proc Natl Acad Sci U S A*. 2014;111(13):4874-4879. doi:10.1073/pnas.1322611111
16. Pesce F, Newcombe EA, Seiffert P, et al. Assessment of models for calculating the hydrodynamic radius of intrinsically disordered proteins. doi:10.1101/2022.06.11.495732
